# Supplementary material for: Isoform‐specific localization of DNMT3A regulates DNA methylation fidelity at bivalent CpG islands
Source: EMBO J. 2017 Oct 26;36(23):3421–34. doi: 10.15252/embj.201797038 (PMC5709737; doi:10.15252/embj.201797038)
Supplement: Supplementary file 1 — Appendix [file EMBJ-36-3421-s001.pdf]

Isoform-specific localization of DNMT3A regulates DNA methylation fidelity at bivalent CpG islands

Massimiliano Manzo[1,2], Joël Wirz[1], Christina Ambrosi[1,2], Rodrigo Villaseñor[1], Bernd Roschitzki[3], and Tuncay Baubec[1]

Affiliations:

1) Department of Molecular Mechanisms of Disease, University of Zurich, Winterthurerstrasse 190, 8057 Zurich, Switzerland

2) Molecular Life Sciences PhD Program of the Life Sciences Zurich Graduate School, University of Zurich, Winterthurerstrasse 190, 8057 Zurich, Switzerland

3) Functional Genomics Center Zurich, ETH and University of Zurich, Winterthurerstrasse 190, 8057 Zurich, Switzerland

Contact:

tuncay.baubec@uzh.ch, Winterthurerstrasse 190, 8057 Zurich, Switzerland, +41 44 635 5438

Contents:

10 Appendix Figures and Legends,

2 Appendix Tables

Appendix Supplementary Methods

Appendix References

Appendix Figure S1

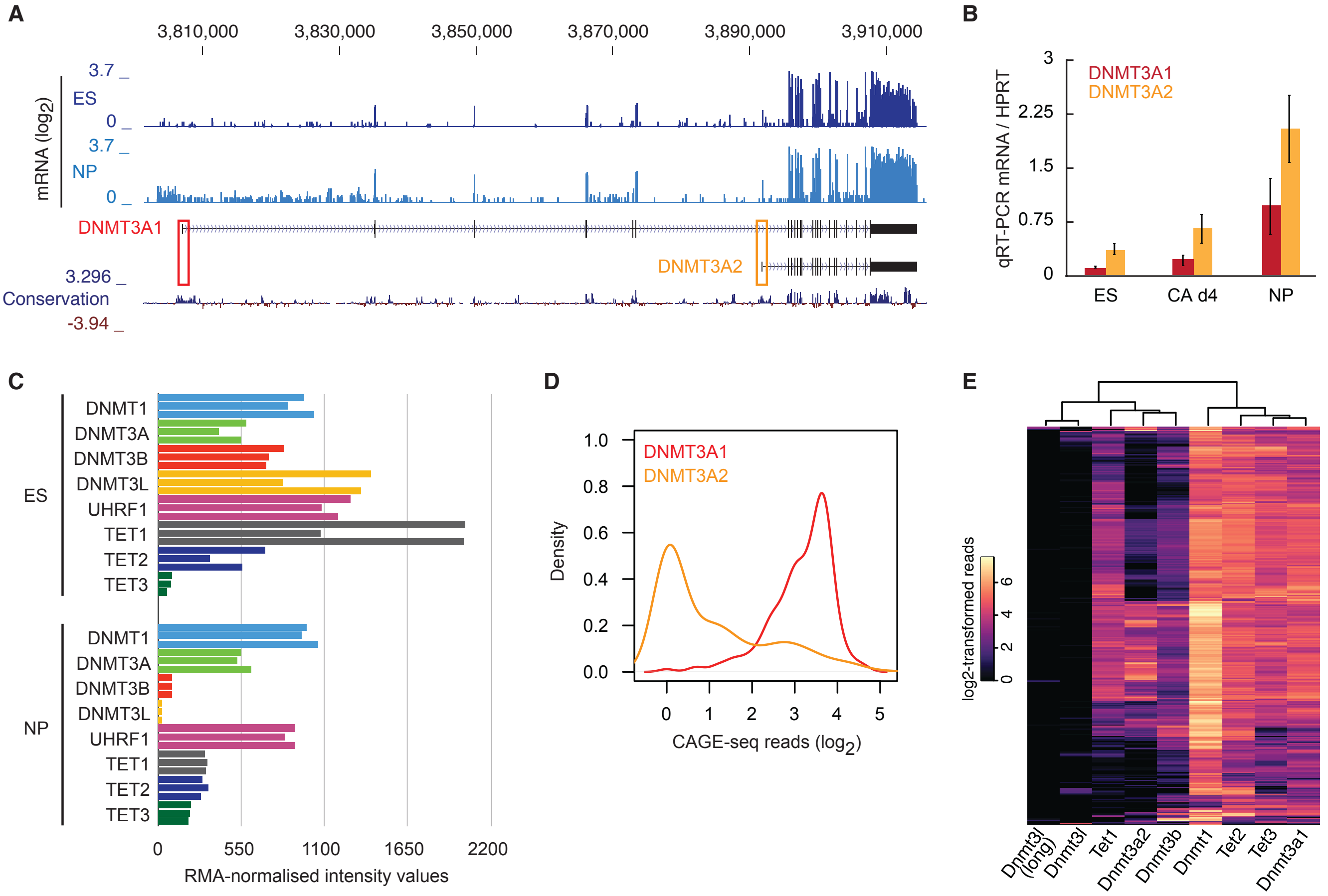

A *Dnmt3a* gene model indicating the promoters and transcripts of the two DNMT3A isoforms, annotated as DNMT3A1 and DNMT3A2. Mammalian conservation obtained from UCSC is shown below. Top tracks: RNA-seq data (library-normalized log_2-transformed reads/100bp) indicating expression from the *Dnmt3a* gene in mouse embryonic stem (ES) and neuronal progenitor (NP) cells. The promoter regions used to extract isoform-specific GAGE-seq reads for DNMT3A1 and DNMT3A2 are highlighted as red and orange boxes, respectively.

B Reverse-transcription qPCR measurement for DNMT3A1 and DNMT3A2 expression during neuronal differentiation. Relative expression of the DNMT3A isoforms has been calibrated to HPRT expression in the same tissues. CA d4 indicates cellular aggregates 4 days after LIF withdrawal. **C** Affymetrix microarray (MoGene-v1) results from ES and NP cells indicate expression levels of various proteins involved in the regulation of DNA methylation. Shown are RMA-normalized intensity values. Data was obtained from (Tiwari *et al,* 2012).

D Density plots indicating DNMT3A1 expression in multiple mouse tissues and developmental stages (red) measured by the abundance of CAGE-seq reads in the respective promoter. DNMT3A2 expression (orange) is restricted to a limited number of tissues. 389 datasets obtained from (FANTOM5 consortium 2014) were used to calculate densities.

E Hierarchical clustering of DNA methylation-relevant genes based on promoter-associated CAGE-seq transcripts (where available). 389 datasets for each gene promoters were used to calculate the heat map. Shown are log_2-transformed and library-normalized CAGE-seq read counts. Only promoters were used that contained read counts in any of the samples and neighboring promoters were collapsed.

Appendix Figure S2

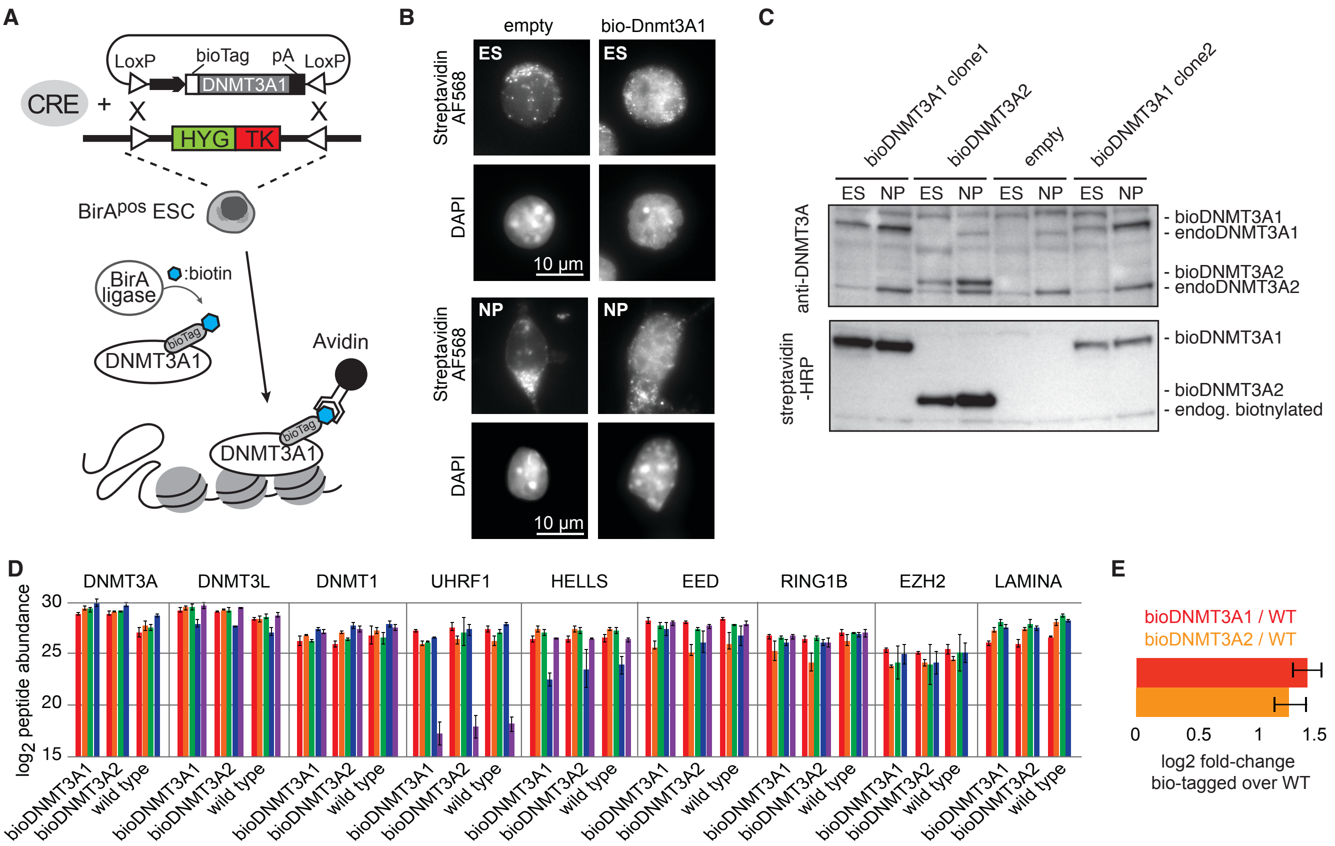

A Outline of the recombinase-assisted mapping of biotinylated proteins (RAMBiO, Baubec *et al,* 2013) procedure. cDNA encoding for the DNMT3A1 protein is introduced to a defined region in the genome using recombinase-mediated cassette exchange (RMCE). Expressed DNMT3A1 proteins are biotinylated on a short amino acid tag by the bacterial Bir-A ligase that is stably expressed in the same cell line. ChIP using streptavidin allows stringent washes and reproducible detection of DNMT3A1 binding in the mouse genome.

B Immunofluorescence detection of biotinylated DNMT3A1 in mouse ES and neuronal progenitor cells by streptavidin fused to AF568 reveals homogenous nuclear localization. Wild type cells (empty) are used as controls. Scale bar: 10 µm.

C Immunoblot detection of endogenous and biotinylated DNMT3 proteins and isoforms from nuclear extracts obtained from mouse ES and neuronal progenitor cells expressing biotin-tagged DNMT3A isoforms from the RMCE site. The two independently-derived clones for biotin-tagged DNMT3A1 which were used in this study are shown. Clone 1 shows higher protein expression compared to clone 2. Detection was performed using antibodies against DNMT3A (top) or HRP-conjugated streptavidin (bottom blot). The signal from endogenous biotinylated proteins serves as loading control.

D Parallel reaction monitoring (PRM) was applied to quantify protein levels in nuclear extracts of wild type cells and cells expressing additional copies of biotin-tagged DNMT3A isoforms. Four independent measurements were performed for four to five unique peptides from the indicated proteins. Shown are the $\log_2$-transformed peptide abundances of the respective proteins in the analyzed cell lines. Note that this analysis does not distinguish endogenous from transgene proteins and the abundance values reflect the total amount of both tagged and endogenous proteins in the cell. The higher-expressing bioDNMT3A1 clone1 was utilized in these experiments.

E Log_2-fold increase in DNMT3A peptide abundance in ES cells expressing biotin-tagged DNMT3A transgenes over wild type calculated based on all DNMT3A peptides measured by PRM. Error bars represent the standard error inferred from four independent replicates.

Appendix Figure S3

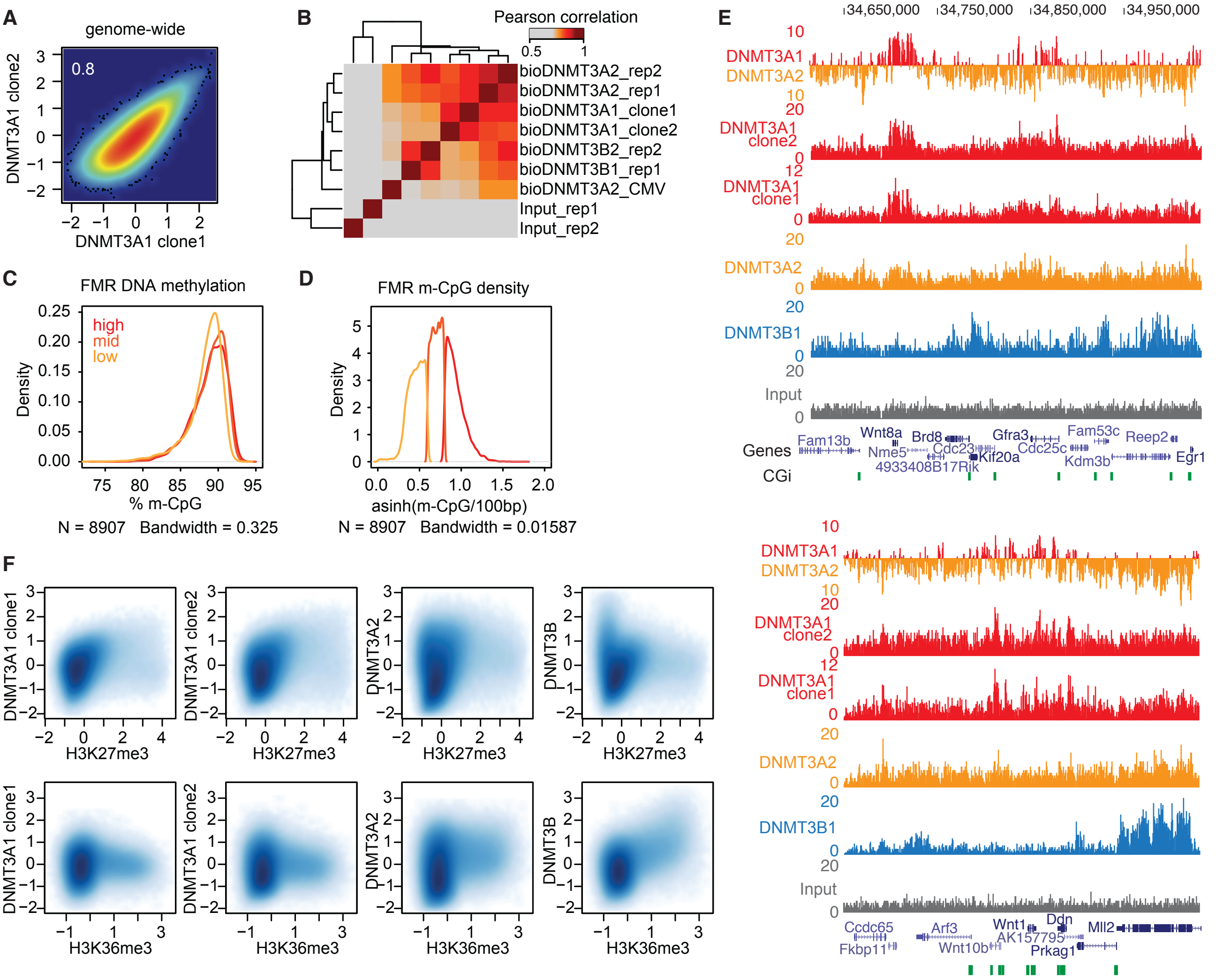

A Scatter plot comparing enrichment of biotinylated DNMT3A1 proteins in the two independently-derived ES cell clones expressing DNMT3A1 from the RMCE site. Pearson's correlation coefficient is shown.

B Cross-correlation analysis of biotinylated *de novo* DNA methyltransferases and input samples using $\log_2$-transformed reads calculated over 1 kb-sized tiles covering the entire genome. Shown are ChIP-seq datasets obtained from two independent DNMT3A1 clones (this study), two independent replicates each from the same clone of DNMT3A2 and DNMT3B1, and one sample obtained from a cell line with low level expression of bio-DNMT3A2 (bioDNMT3A2_CMV) from (Baubec *et al,* 2015). DNMT3A2 shows more similar binding to DNMT3A1 and DNMT3B, while the two latter proteins differ more to each other in genomic binding, reflecting their individual preferences to specific genomic sites. Protein specific binding to the genome is not influenced by expression levels of biotinylated proteins. This is observed from the correlation between bioDNMT3A2_CMV and the DNMT3A2 datasets with higher expression, and also by the good correlation between the DNMT3A1 clones that differ in bioDNMT3A1 expression levels (Appendix Fig S2).

C, D Density plot indicating similar DNA methylation percentage in the three FMR fractions binned by DNA methylation density utilized in Fig 1C. D DNA methylation density shown for the same fractions as in C.

E Genome browser view exemplifying differences in binding between the *de novo* methyltransferases. Shown are read counts per 100 bp for ChIP-seq and input samples. Tracks from two independent DNMT3A1 clones and one track indicating differences between the DNMT3A isoforms are shown. Gene models and CpG islands obtained from the UCSC genome browser are indicated.

F Scatter plots showing genome-wide correlations between DNMT3 proteins and H3K27me3 or H3K36me3 marks over 1 kb-sized tiles covering the entire genome.

Appendix Figure S4

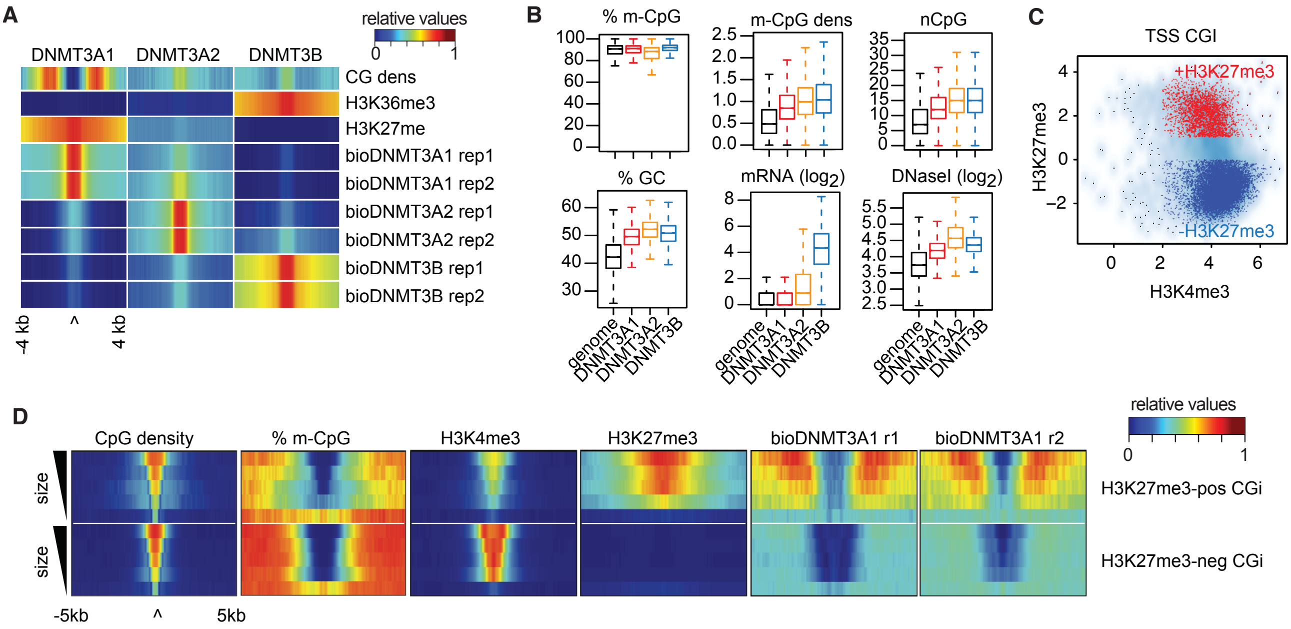

A Heat map representation of average densities calculated for DNMT3 replicates, CpG, H3K27me3 and H3K36me3 signals at sites exclusively bound by DNMT3A1, DNMT3A2 or DNMT3B, respectively.

B Box plots indicating DNA methylation, DNA sequence features, mRNA and DNaseI accessibility at sites exclusively bound by the individual *de novo* DNMTs. Boxes denote the inter-quartile range (IQR) and whiskers 1.5 x IQR.

C Scatter plot displaying H3K27me3 and H3K4me3 enrichments over input at CpG island promoters. Red and blue dots denote the bivalent and H3K4me3-only CpG islands that have been selected for further analyses.

D Heat map representation of DNMT3A1 binding and other features around H3K27me3-positive and -negative CpG islands, binned into five equally-sized groups based on the annotated CpG island size.

Appendix Figure S5

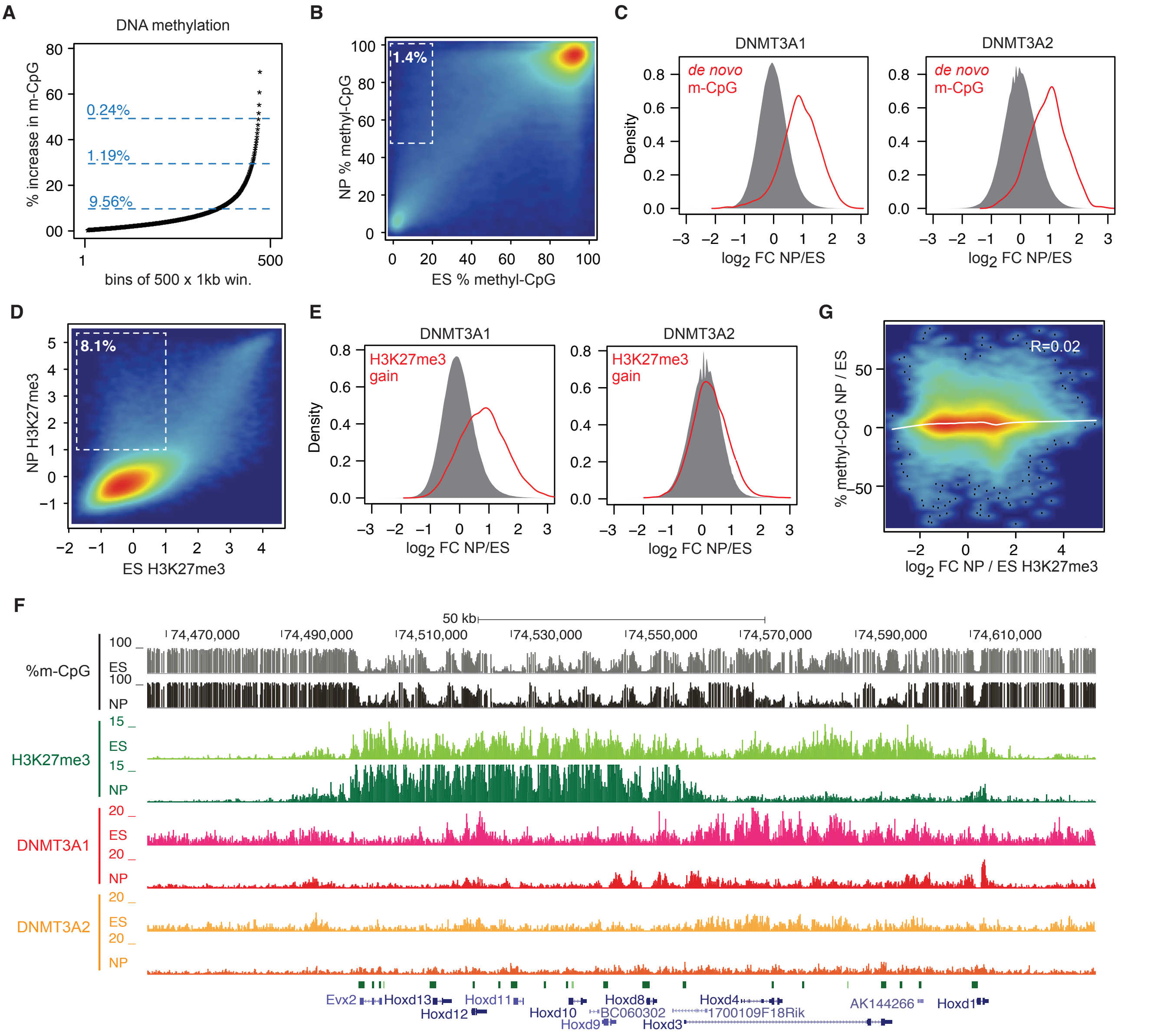

A Genomic regions ranked based on *de novo* DNA methylation during ES to NP differentiation. Shown are median NP/ES DNA methylation values calculated over 500 x 1 kb windows ranked by the level of *de novo* methylation. Blue lines denote % of genomic windows that gain the indicated *de novo* methylation in NPs.

B Scatterplot indicating DNA methylation in ES and NP cells calculated at 1 kb windows covering the entire genome. Dashed box highlights regions selected as *de novo* methylated (used in C) and the fraction of the genome represented by this set that contains 23,725 CpGs.

C Density plot indicating the log_2-fold change in DNMT3A1 and DNMT3A2 binding during neuronal differentiation at all genomic regions (gray) and at *de novo* methylated regions (red).

D Scatterplot indicating H3K27me3 log_2-enrichment over input in ES and NP cells calculated using 1 kb windows covering the entire genome. Dashed box highlights regions selected as gaining H3K27me3 in NPs (used in E) and the fraction of the genome represented by this set.

E Density plot indicating the log_2-fold change in DNMT3A1 and DNMT3A2 binding during neuronal differentiation at all genomic regions (gray) and at regions gaining H3K27me3 (red).

F Genome browser example for regions that change DNMT3A1 binding and DNA methylation according to changes in H3K27me3. Shown are read counts per 100 bp for ChIP-seq datasets and percentage of DNA methylation per individual CpGs.

G Scatterplot comparing dynamics in DNA methylation and H3K27me3 during neuronal differentiation using 1 kb windows covering the entire genome. Pearson's correlation coefficient is shown.

Appendix Figure S6

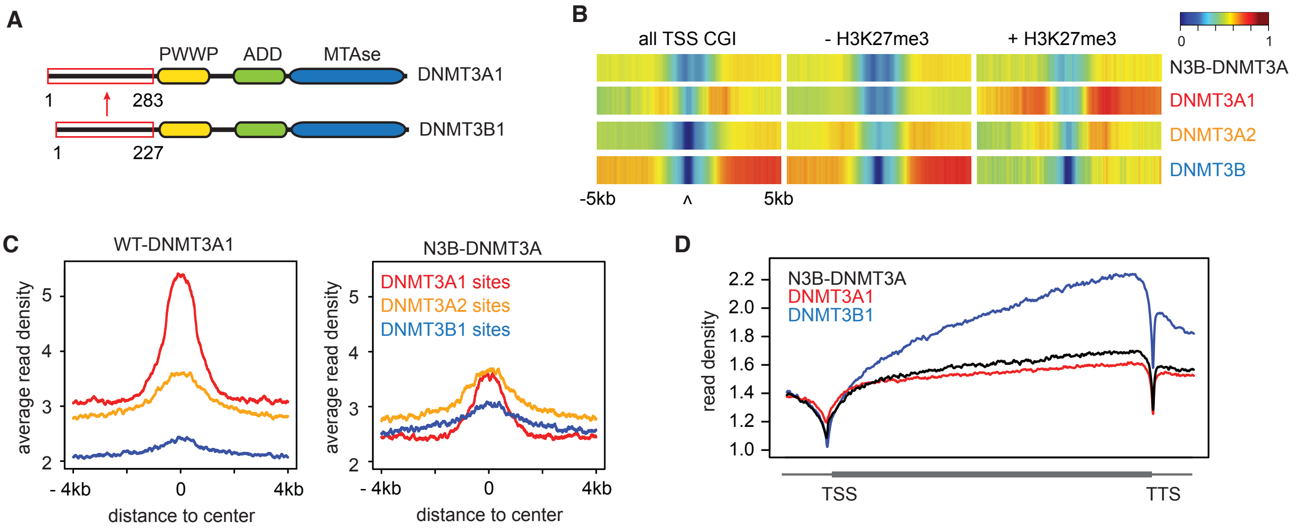

A Diagram indicating the strategy used to engineer the chimeric N3B-DNMT3A protein. The N-terminal part of DNMT3A1 (AA 1 to 283) was replaced by the N-terminal part of DNMT3B (AA 1 to 227).

B N3B-DNMT3A lacks the preferential localization to bivalent CpG island promoters. Shown are heat map density profiles for DNMT3 protein binding around all promoter-associated CpG islands, and CpG promoters separated by H3K27me3.

C N3B-DNMT3A fails to bind sites preferentially enriched for WT DNMT3A1. Shown are average density profiles for WT DNMT3A1 and N3B-DNMT3A around sites exclusively bound by DNMT3A1, DNMT3A2 or DNMT3B in red, orange and blue, respectively.

D Addition of the DNMT3B N-terminal part to DNMT3A does not recruit the chimeric N3B-DNMT3A protein to transcribed gene bodies. Shown are average density profiles for WT DNMT3A1, WT-DNMT3B and N3B-DNMT3A around genes.

Appendix Figure S7

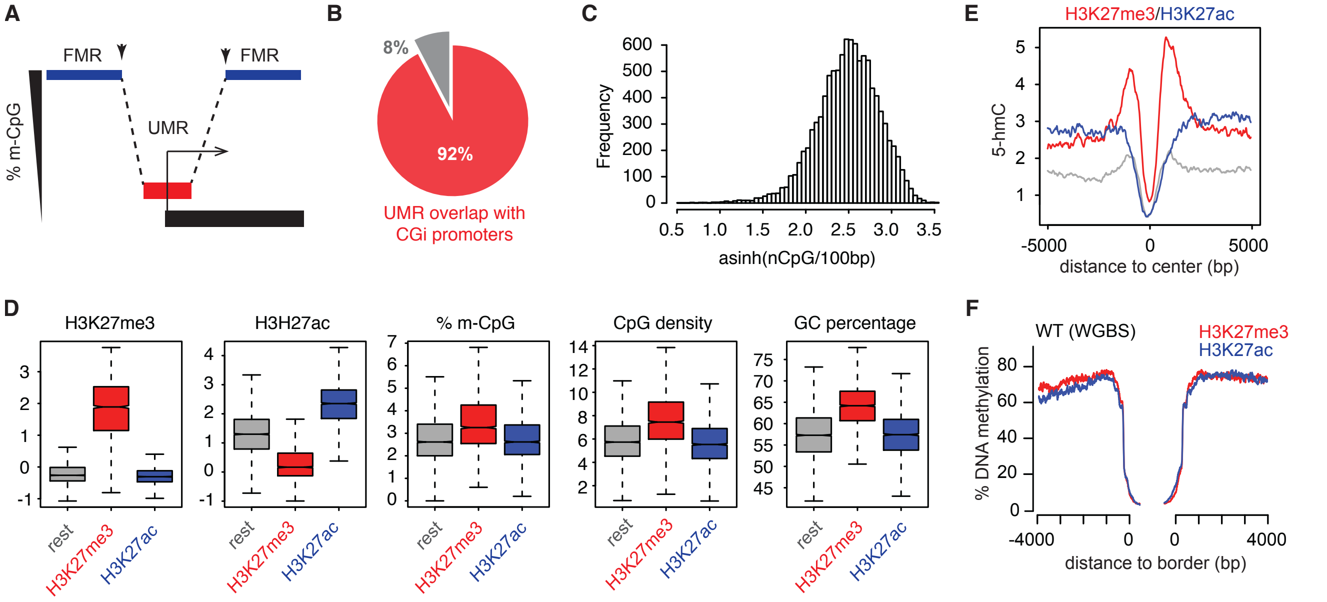

A Schematic representation of FMR and UMR transitions based on DNA methylation in ES cells from Stadler *et al*, 2011. UMRs overlapping promoters were selected and oriented according to the gene orientation. Arrowheads indicate borders used in Fig 5 and 6.

B Overlap between promoter-associated UMRs and promoter CpG islands.

C Histogram indicating elevated CpG density at selected UMRs. Shown are numbers of CpGs per 100bp calculated for each UMR.

D Box plots indicating H3K27me3, H3K27ac, methylation and CpG properties of UMRs selected based on H3K27me3 (red) and H3K27ac (blue) levels (top 25% ranked). Remaining UMRs are shown in gray. Boxes denote the inter-quartile range (IQR) and whiskers 1.5 x IQR.

E Average density plots for 5-hmC centered around UMRs selected based by H3K27me3 (red) or H3K27ac (blue) levels indicate elevated 5hmC at Polycomb UMR borders.

F Average density plots for DNA methylation in wild type cells (WGBS) around H3K27me3 (red) or H3K27ac (blue) UMRs indicate no visible differences in DNA methylation around these regions.

Appendix Figure S8

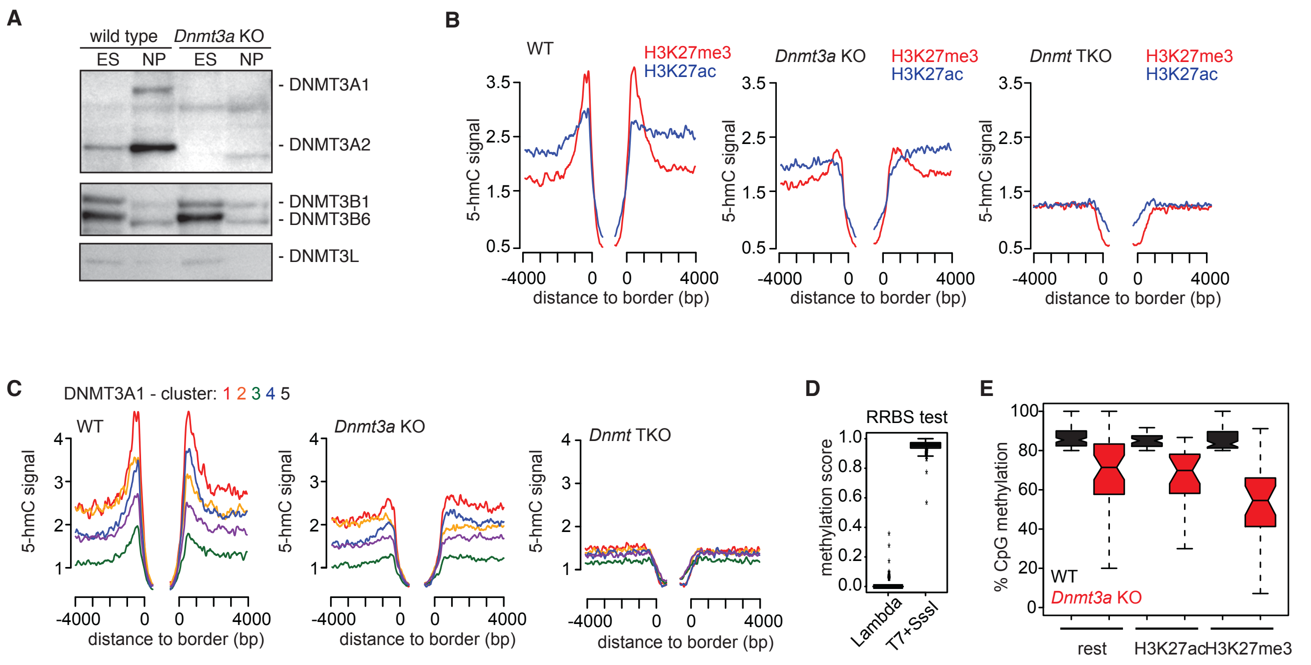

A Immunoblot detection of DNMT3A, DNMT3B and DNMT3L in wild type and *Dnmt3a* KO ES and NP cells. *Dnmt3a* KO cells were generated by CRISPR/Cas9 with dual guides targeting exon seven common for both isoforms.

B Average density profiles centered at the borders between FMR and UMRs showing 5-hmC signals in wild type, *Dnmt3a* KO ES cells indicating predominant loss of 5-hmC at H3K27me3-positive UMR borders in absence of DNMT3A. Red indicates the signal around H3K27me3-positive UMRs and blue around H3K27ac-positive UMRs. *Dnmt*-TKO ES cells serve as background control for the 5-hMeDIP-seq experiments.

C Same data as in B, but using clusters defined based on DNMT3A1 binding (see Fig EV2).

D Bisulphite conversion test for RRBS showing measured CpG methylation for spiked-in unmethylated lambda phage DNA and *Sss*I-methylated T7 phage DNA.

E Box plots indicating reduced DNA methylation at H3K27me3-positive CpG island shores in absence of DNMT3A. Only sites with DNA methylation > 80% in wild type cells were used for this calculation.

Appendix Figure S9

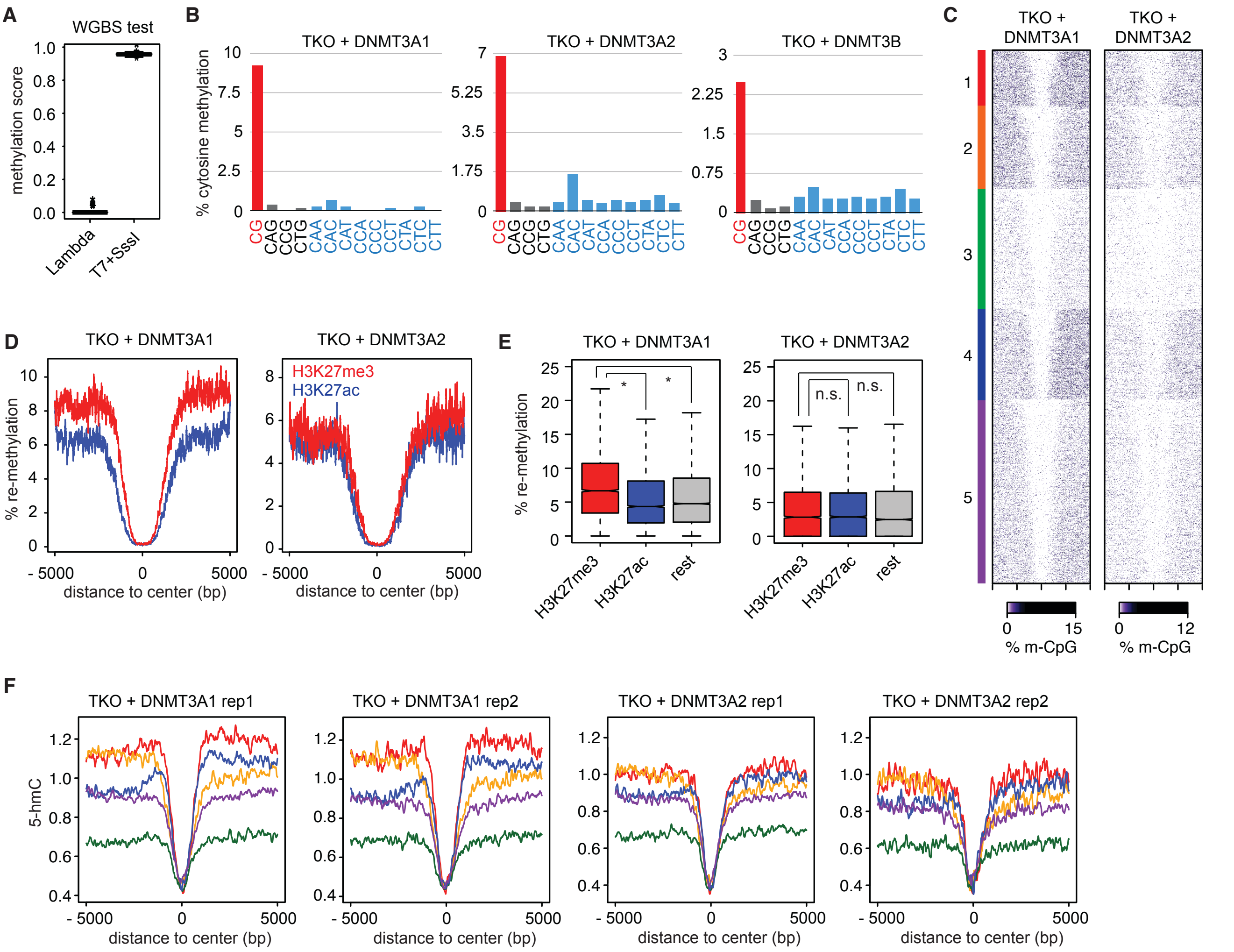

A Bisulphite conversion test for WGBS showing measured CpG methylation for spiked-in unmethylated lambda phage DNA and *Sss*I-methylated T7 phage DNA.

B Bar plots indicating percentage of cytosine methylation measured separately at CpG dinucleotides and various non-CpG sites in TKO cells expressing DNMT3A1, DNMT3A2 or DNMT3B.

C Heat map profiles for all promoter-associated UMRs clustered by DNMT3A1 binding and ranked by size (according to Figure EV2). Shown are *de novo* DNA methylation in TKO cells expressing DNMT3A1 or DNMT3A2. Scales have been adjusted in order to visualize the sparse methylation data obtained from these measurements.

D Average density profiles centered around H3K27me3-positive (red) and H3K27ac-positive (blue) UMRs indicate increased re-methylation of H3K27me3 UMRs in DNMT3A1, but not in DNMT3A2-rescued *Dnmt*-TKO cells.

E Calculated gain in DNA methylation in DNMT3A1 or DNMT3A2-rescued *Dnmt*-TKO cells at UMR borders according to H3K27me3 (red), H3K27ac (blue) and remaining UMRs (gray). Boxes denote the inter-quartile range (IQR) and whiskers 1.5 x IQR. Asterisk denotes p-values < 2.2e -16, and n.s. > 0.5 calculated each between two samples using unpaired t-tests.

F Average density plots indicating 5-hmC gain around DNMT3A1-bound UMR promoters (clustered as in Fig EV2) in TKO cells expressing DNMT3A1 or DNMT3A2. Two independent replicates are shown for each cell line.

Appendix Figure S10

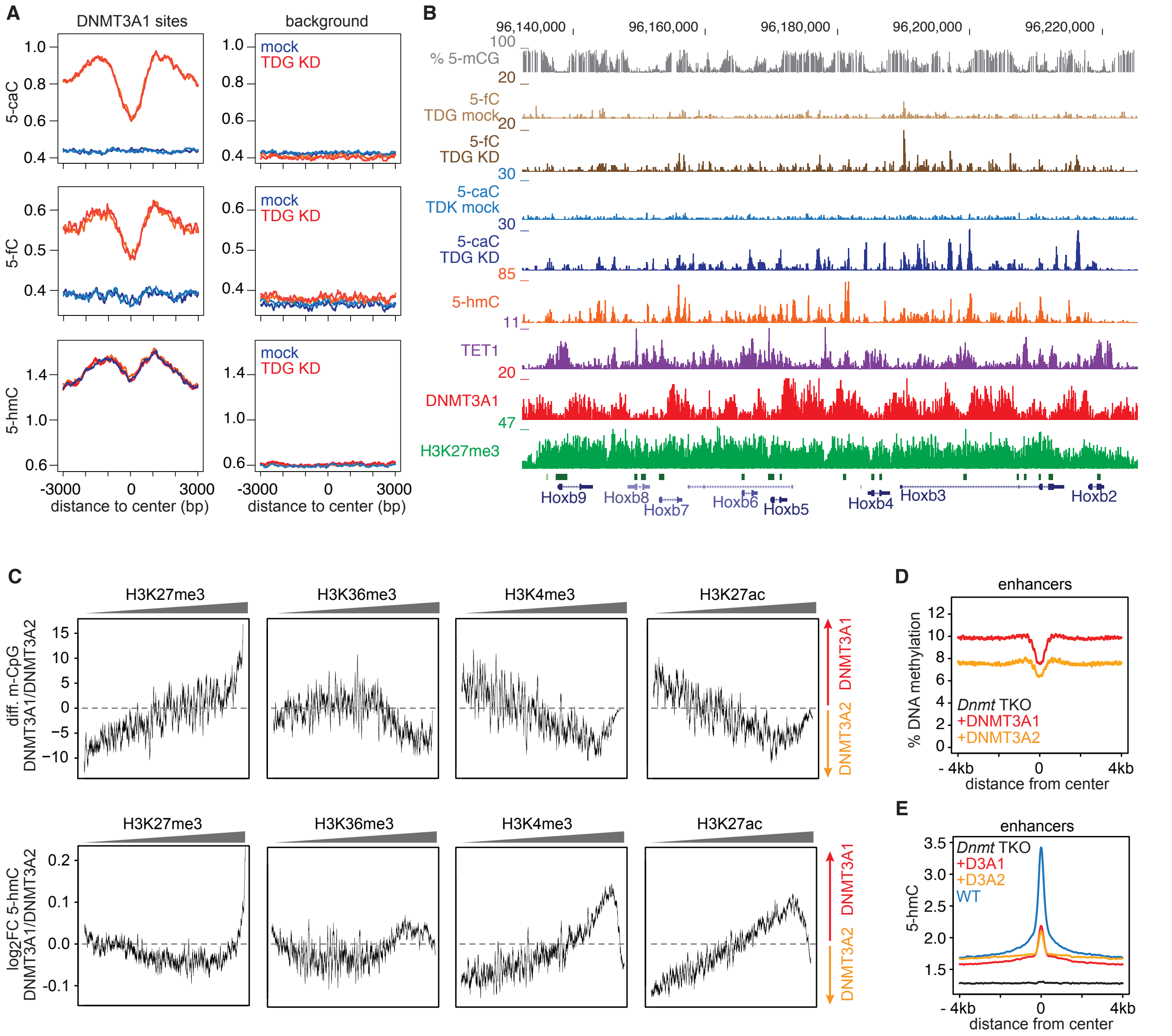

A Average density profiles for 5-caC, 5-fC and 5-hmC in wild type and TDG knock-down ES cells obtained from (Shen *et al,* 2013). Shown are signals around DNMT3A1-bound sites and background genomic sites defined as fully methylated regions (FMR) with low CpG density. KD of TDG leads to increased 5-caC and 5-fC signals around DNMT3A1 binding sites, highlighting elevated turnover of methylated cytosines around these regions.

B Genome browser example for regions that change 5-fC and 5-caC in absence of TDG at sites enriched for DNMT3A1, TET1 and H3K27me3. Shown are read counts per 100 bp for ChIP-seq datasets and percentage of DNA methylation per individual CpGs.

C Plots showing isoform-specific *de novo* DNA methylation activity (top) or $\log_2$ differences in 5-hmC levels (bottom) between *Dnmt*-TKO cells expressing DNMT3A1 and DNMT3A2. Differences were calculated over 1kb-sized genomic tiles covering the entire genome and ranked by enrichment of histone modifications. A sliding window of 200 consecutive tiles was applied for the representation of the data. Only tiles with sufficient coverage in WGBS were utilized for representing the difference in m-CpG.

D Average density plots showing *de novo* DNA methylation activity of DNMT3A1 or DNMT3A2 at enhancers measured in TKO cells expressing the respective enzymes.

E Average density plots showing 5-hmC signals around enhancers in WT cells, *Dnmt*-TKO cells and TKO cells expressing either DNMT3A1 or DNMT3A2.

Appendix Table S1

Sequencing information for datasets provided in this study (GSE96529):

sample name according to GSE96529	experiment type	sequencing type	reads sequenced	uniquely mapped reads
ES_input_rep1	input-seq	single	54.3 mio	39.4 mio
ES_input_rep2	input-seq	single	56.5 mio	40.6 mio
ES_bioDNMT3A1_rep1 (clone1)	bioChIP-seq	single	50 mio	33.9 mio
ES_bioDNMT3A1_rep2 (clone2)	bioChIP-seq	single	84.5 mio	52.9 mio
ES_N3B-DNMT3A	bioChIP-seq	single	42.5 mio	31.1 mio
ES_5hMeDIP_WT_rep1	5hMeDIP-seq	single	38.9 mio	24.6 mio
ES_5hMeDIP_WT_rep2	5hMeDIP-seq	single	20.6 mio	14.5 mio
ES_5hMeDIP_Dnmt3aKO_rep1	5hMeDIP-seq	single	44.7 mio	29.6 mio
ES_5hMeDIP_Dnmt3aKO_rep2	5hMeDIP-seq	single	23.6 mio	15.9 mio
ES_5hMeDIP_DnmtTKO	5hMeDIP-seq	single	39.3 mio	22.3 mio
ES_5hMeDIP_TKO_Dnmt3a1_rep1	5hMeDIP-seq	single	50 mio	23.8 mio
ES_5hMeDIP_TKO_Dnmt3a1_rep2	5hMeDIP-seq	single	16.3 mio	9.3 mio
ES_5hMeDIP_TKO_Dnmt3a2_rep1	5hMeDIP-seq	single	38.5 mio	20.8 mio
ES_5hMeDIP_TKO_Dnmt3a2_rep2	5hMeDIP-seq	single	16.9 mio	7.9 mio

sample name according to GSE96529	experiment type	sequencing type	reads sequenced	CpGs covered *	bisulphite conversion
WT_RRBS	RRBS	single	26.7 mio	970 k	> 95%
Dnmt3aKO_RRBS	RRBS	single	25.4 mio	814 k	> 95%
DnmtTKO_RRBS	RRBS	single	35.8 mio	432 k	> 95%
DnmtTKO+Dnmt3A1_WGBS	WGBS	paired	175.4 mio	9.2 mio	> 98%

*) for RRBS > 20 x coverage; for WGBS > 10 x coverage

Appendix Table S2

External datasets used in this study:

Sample	Reference	Source
ES + NP_mRNA_seq	Tippmann et al., 2012	GSE33252
ES + NP H3K27me3	Tippmann et al., 2012	GSE33252
ES + NP H3K36me3	Tippmann et al., 2012	GSE33252
Affymetrix_MoGeneV1	Tiwari et al., 2012	GSE27245
CAGE-seq data	FANTOM consortium 2014	http://fantom.gsc.riken.jp/5/
ES + NP m-CpG_WGBS	Stadler et al., 2011	GSE30206
ES_bioDNMT3A2_rep1	Baubec et al., 2015	GSE57413
ES_bioDNMT3A2_rep2	Baubec et al., 2015	GSE57413
ES_bioDNMT3B1_rep1	Baubec et al., 2015	GSE57413
ES_bioDNMT3B1_rep2	Baubec et al., 2015	GSE57413
CMV_bioDNMT3A2	Baubec et al., 2015	GSE57413
WGBS_TKO_DNMT3A2	Baubec et al., 2015	GSE57413
WT_ES_H3K4me3	ENCODE consortium, 2012	GSE49847
WT_ES_H3K4me1	ENCODE consortium, 2012	GSE49847
WT_ES_H3K27ac	ENCODE consortium, 2012	GSE49847
WT_DHS_seq	ENCODE consortium, 2012	GSE37074
H3K9me3	Marks et al., 2012	GSE23943
H3K27me1	Ferrari et al., 2014	GSE39496
H2Aub	Brookes et al., 2012	GSE34520
RNA Pol2 S2P, S5P	Brookes et al., 2012	GSE34520
ES + NP 5-hMeDIP	Feldmann et al., 2013	GSE39739
Suz12, Ring1B	Morey et al., 2013	GSE42466
TET1	Wu et al., 2011	GSE26833
TKO + DNMT3x H3K27me3	King et al., 2016	GSE77004
WT + TKO DHS	Domcke et al., 2015	GSE67867
WT + TKO RNA-seq	Domcke et al., 2015	GSE67867
WT + TDG KD 5-caC, 5-fC	Shen et al, 2013	GSE42250

Appendix Supplementary Methods

RNA isolation, cDNA synthesis and qPCR

RNA was isolated from mouse ESCs, cellular aggregates at day 4 and NPCs using the RNeasy mini kit (Qiagen). Genomic DNA was removed through on-column digestion with DNase I for 15 min at room temperature. cDNA was synthesized from 2 µg isolated RNA with SuperScript III First-Strand Synthesis (Invitrogen) for 60 min at 50 °C using random hexamer primers, followed by heat-inactivation for 10 min at 70 °C. Residual RNA was digested with 2 U RNase H for 20 min at 37 °C. Target sequences were quantified by real-time qPCR analysis using a KAPA SYBR FAST qPCR Kit (Kapa Biosystems) on a QuantStudio 5 Real-Time PCR System (Applied Biosystems). Comparative quantification (ddCt) was used to determine transcript levels relative to HPRT. Each sample was run in technical triplicates. Following primers were used: Hprt: GG ACCTCTCGAAGTGTTGGA and CAACTTGCGCTCATCTTAGGC; Dnmt3A_universal: TGAGACTCTCCAGA GGCCTGGT; Dnmt3A1_specific: ACCCATGCCAAGACTCACCTTC; Dnmt3A2_specific: CACACCTGAGCT GTACTGCAGAGGGG.

Nuclear extract preparation and Immunoblotting

Cell pellets were resuspended in 1 mL 10 mM EDTA, 10 mM KCl, 10 mM HEPES, 1 mM Dithiotreitol, 1 × Protease Inhibitor cocktail, 0.1% NP-40 (Nonidet P40), incubated for 10 min on ice followed by centrifugation for 10 min at 12,000 × g at 4°C. Supernatants were removed and the pellets were resuspended in 100 µL 20 mM HEPES, 0.4 M NaCl, 1 mM EDTA, 1% glycerol, 1 mM Dithiotreitol, 1 × Protease Inhibitor cocktail. Nuclear pellets were incubated for 2 h at 4°C shaking at 2,000 rpm to resuspend. Debris was removed by centrifugation for 10 min at 20,000 × g at 4°C. Supernatant contained nuclear protein fraction. Equal amounts of protein extracts were resuspended in denaturing and loading buffer, and separated on precast gels according to manufacturers instructions (NuPAGE). Proteins were transferred to PVDF membranes (Amersham, 0.22 µm) by Western blotting. Membranes were blocked with 5% milk for antibody detection and with 5% BSA for streptavidin detection. Following antibodies were used: anti-DNMT3A (IMG-268A), anti-DNMT3B (IMG-184A), anti-DNMT3L (ab3493) and Streptavidin-HRP (Pierce).

Immunofluorescence

Cells were cultivated on poly-L-lysine-coated coverslips, fixed for 10 min in 3% PFA and permeabilized in 0.1% NaCitrate and 0.1% Triton X-100. Samples were blocked for 30 min with 0.1% Tween20, 3% BSA (w/v) and 10% normal goat serum in PBS, and detection was performed with Streptavidin-AF568 (Invitrogen) over night at 4 °C. Coverslips were washed with 0.1% Tween-20 and 0.25% BSA (w/v) in PBS. Nuclei were counterstained with DAPI. Images were acquired using a Zeiss Z1 epifluorescence microscope.

Parallel Reaction Monitoring

Dissolved samples were injected by an Easy-nLC 1000 system (Thermo Scientific) and separated on an EasySpray-column (75 µm x 500 mm) packed with C18 material (PepMap, C18, 100 Å, 2 µm, Thermo Scientific). The column was equilibrated with 100% solvent A (0.1% formic acid (FA) in water). Peptides were eluted using the following gradient of solvent B (0.1% FA in ACN): 5% B for 2min; 5-25% B in, 90 min; 25-35% B in 10 min; 35-99% B in 5 min at a flow rate of 0.3 µl/min. High accuracy mass spectra were acquired with an Orbitrap Fusion (Thermo Scientific) that was operated in targeted MS/MS mode. The method contained a full MS scan from 300-1500 m/z analyzed in the Orbitrap with a resolution of 60,000 @ 200 m/z. Maximum injection time was set to 50 ms and a AGC target value of 4e5 was used. The targeted MS2 spectra were

recorded from 200-1800 m/z in the Orbitrap using following parameter: AGC target 5e4, maximum injection time 54 ms, resolution 30,000 @ 200 m/z, quadrupole isolation, isolation window 1.6m/z, HCD 30% fragmentation energy, loop count 15. The target inclusion list was prepared using Skyline 3.7 (MacLean et al., 2010) and based on the predicted retention time of each peptide the precursor list was ordered by retention time and divided in 2 lists containing an equal number of targets. Targets within ± 5min of the separation point were transferred in the respective other list in order to avoid peak splitting. The first target list of 45 precursors was analyzed from 0-50min and the second target list from 50-112 min using the same MS2 parameter. The recorded MS RAW-data were imported into Skyline 3.7 and all peak groups were revised manually. For the group comparison we have used MSstats_3.7.3 (Choi et al., 2014) with the following settings: normalization – equalized medians; missing peaks are allowed, using high quality features were selected, and WT was set as control group.

Appendix References:

Brookes, E., de Santiago, I., Hebenstreit, D., Morris, K. J., Carroll, T., Xie, S. Q., et al. (2012). Polycomb Associates Genome-wide with a Specific RNA Polymerase II Variant, andRegulates Metabolic Genes in ESCs. Stem Cell, 10(2), 157–170.

Choi M., Chang, C.Y., Clough, T., Broudy, D., Killeen, T. et al. (2014). MSstats: an R package for statistical analysis of quantitative mass spectrometry-based proteomic experiments, Bioinformatics, 30, 17, 2524–2526

Ferrari, K. J., Scelfo, A., Jammula, S., Cuomo, A., Barozzi, I., Stützer, A., et al. (2014). Polycomb-dependent H3K27me1 and H3K27me2 regulate active transcription and enhancer fidelity. Molecular Cell, 53(1), 49–62.

Morey, L., Aloia, L., Cozzuto, L., Benitah, S. A., & Di Croce, L. (2013). RYBP and Cbx7 Define Specific Biological Functions of Polycomb Complexes in Mouse Embryonic Stem Cells. CellReports, 3(1), 60–69.

Tippmann, S. C., Ivanek, R., Gaidatzis, D., Schöler, A., Hoerner, L., van Nimwegen, E., et al. (2012). Chromatin measurements reveal contributions of synthesis and decay to steady-state mRNA levels. Molecular Systems Biology, 8.

Tiwari, V. K., Burger, L., Nikoletopoulou, V., Deogracias, R., Thakurela, S., Wirbelauer, C., et al. (2012). Target genes of Topoisomerase IIβ regulate neuronal survival and are defined by their chromatin state. Pnas, 109(16), E934–43.

Yue, F., Cheng, Y., Breschi, A., Vierstra, J., Wu, W., Ryba, T., et al. (2014). A comparative encyclopedia of DNA elements in the mouse genome. Nature, 515(7527)
