# Supplementary material for: Isoform‐specific localization of DNMT3A regulates DNA methylation fidelity at bivalent CpG islands
Source: EMBO J. 2017 Oct 26;36(23):3421–34. doi: 10.15252/embj.201797038 (PMC5709737; doi:10.15252/embj.201797038)
Supplement: Supplementary file 2 — Expanded View Figures PDF [file EMBJ-36-3421-s002.pdf]

Expanded View Figures

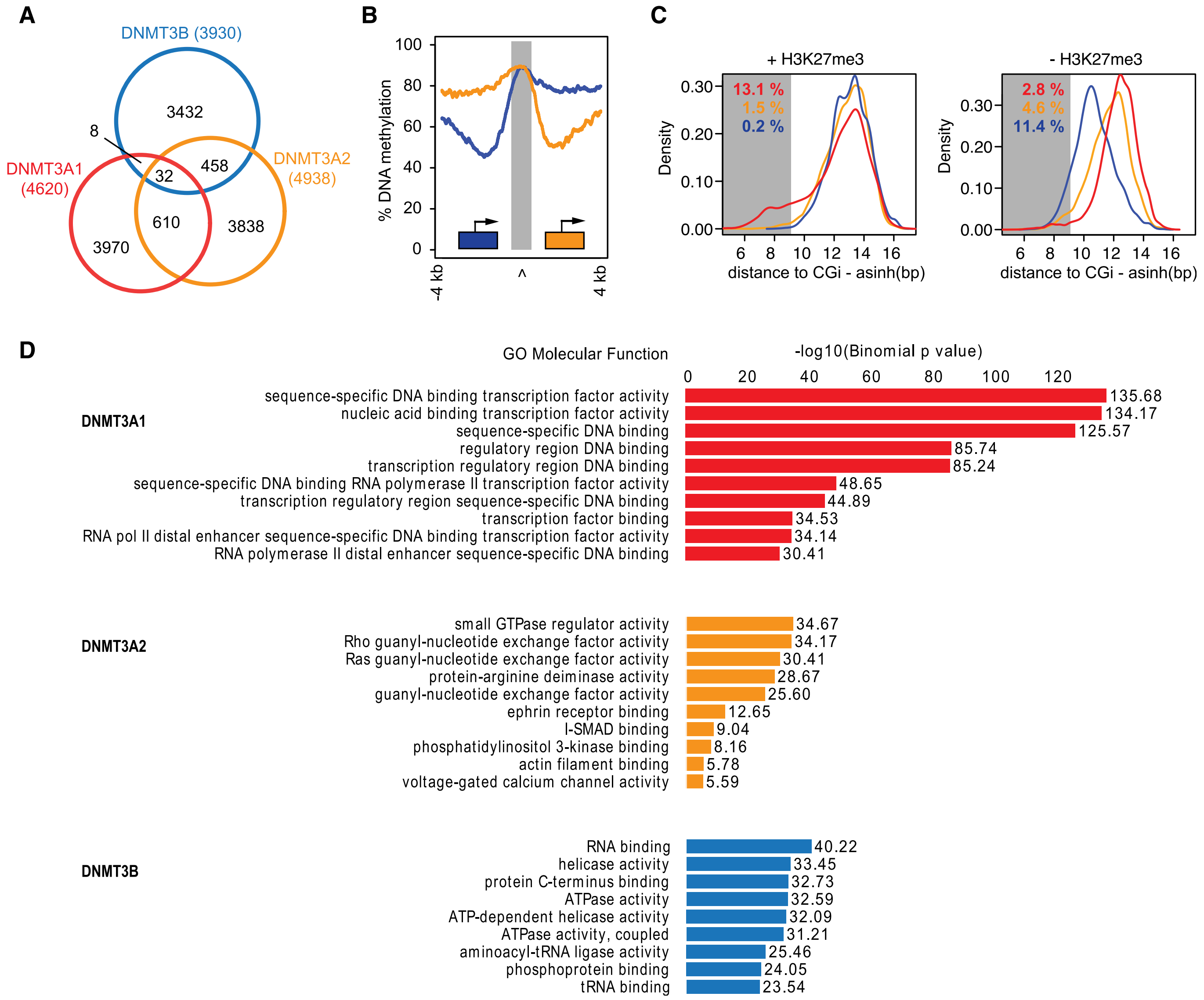

Figure EV1. DNMT3A1 localization around Polycomb CpG islands.

A Venn diagram indicating overlapping and individual binding sites for the *de novo* DNA methyltransferases identified from 1-kb-sized tiles covering the entire genome.

B Averaged DNA methylation % around DNMT3A1-bound sites (in gray) according to their position upstream (5′) or downstream (3′) of neighboring CpG island promoters. Blue line indicates DNMT3A1 sites downstream of the 3′-end, and orange line upstream of the 5′-end of CGI promoters.

C Density plots indicating the distance between *de novo* DNA methyltransferase-enriched sites (red: DNMT3A1, orange: DNMT3A2, blue: DNMT3B) and CpG island promoters separated by H3K27me3. Shaded area indicates a distance shorter than 5 kb, and the percentage of bound sites within that distance is indicated for all DNMT3 proteins in the corresponding color.

D GREAT (McLean *et al*, 2010) analysis of DNMT3-binding sites indicating the enriched Molecular Function Gene Ontology term of nearby genes. DNMT3A1 frequently associates with or near promoters of DNA sequence-dependent transcriptional regulators.

© 2017 The Authors

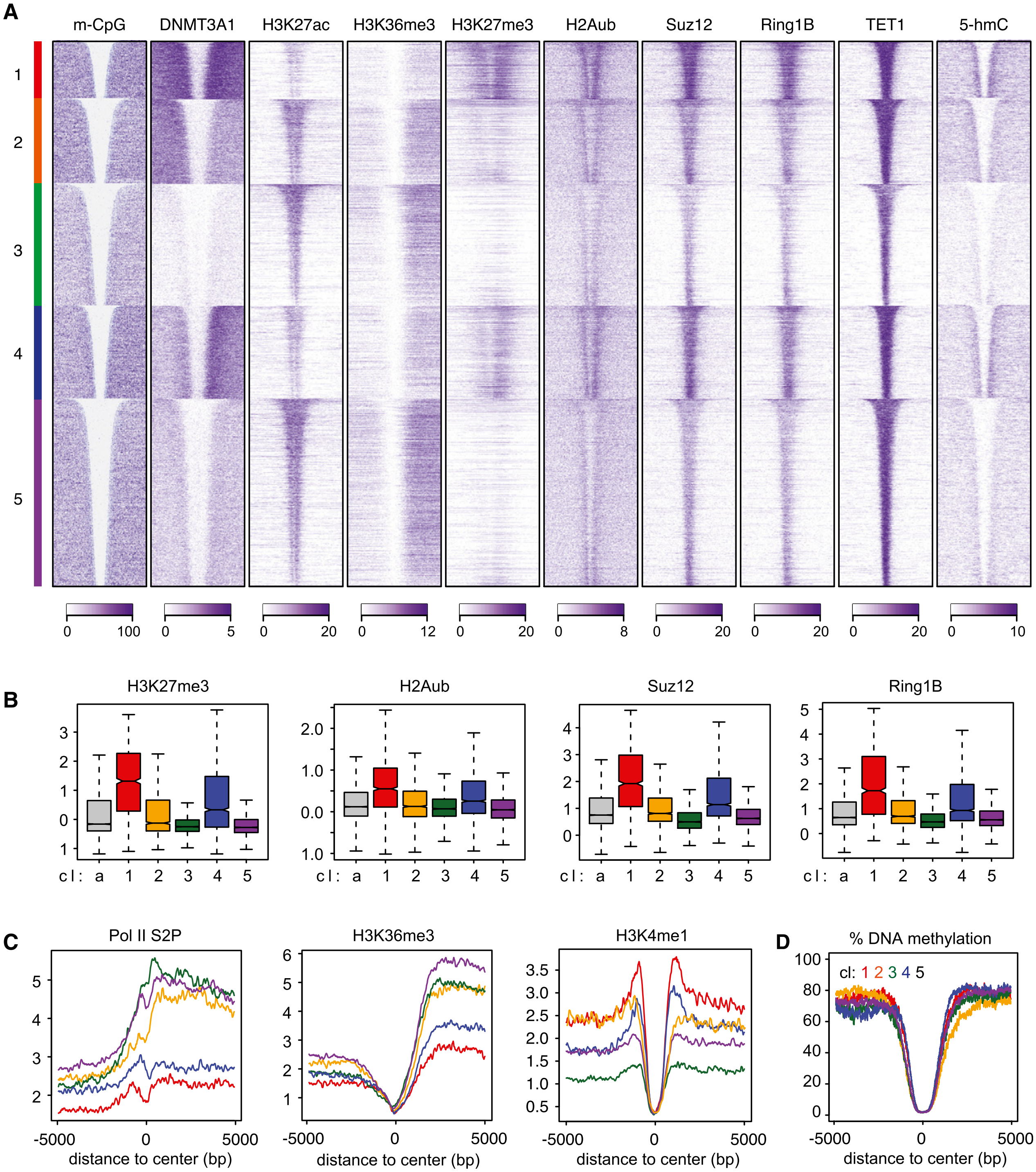

Figure EV2. Preferential DNMT3A1 binding to CpG island shores coincides with elevated Polycomb activity and TET-mediated oxidation.

A Heat map profiles for all promoter-associated UMRs clustered by DNMT3A1 binding and ranked by size. Shown are DNMT3A1 and various chromatin features clustered and ordered according to DNMT3A1 binding into five clusters. Note that clusters 1 and 4 contain the highest enrichment for DNMT3A1, whereas DNMT3A1 binding in cluster 2 is moderate and occurs upstream of the UMR.

B Box plots indicating enrichment of Polycomb marks and proteins within UMRs at clusters preferentially bound by DNMT3A1. Shown are log_2-enrichments over input. Boxes denote the inter-quartile range (IQR) and whiskers 1.5 × IQR. Notches display the confidence interval around the median.

C Average density plots indicate elevated transcriptional activity at clusters 2, 3, and 5. Shown are binding profiles for RNA polymerase II phosphorylated on serine 2 and H3K36me3 as markers of transcriptional activity. In addition, H3K4me1 that scales similarly to 5-hmC at DNMT3A1-enriched clusters is shown (compare to Fig 5C).

D Average density plots for DNA methylation in wild-type cells measured by WGBS around UMRs clustered based on DNMT3A1 binding.

 © 2017 The Authors

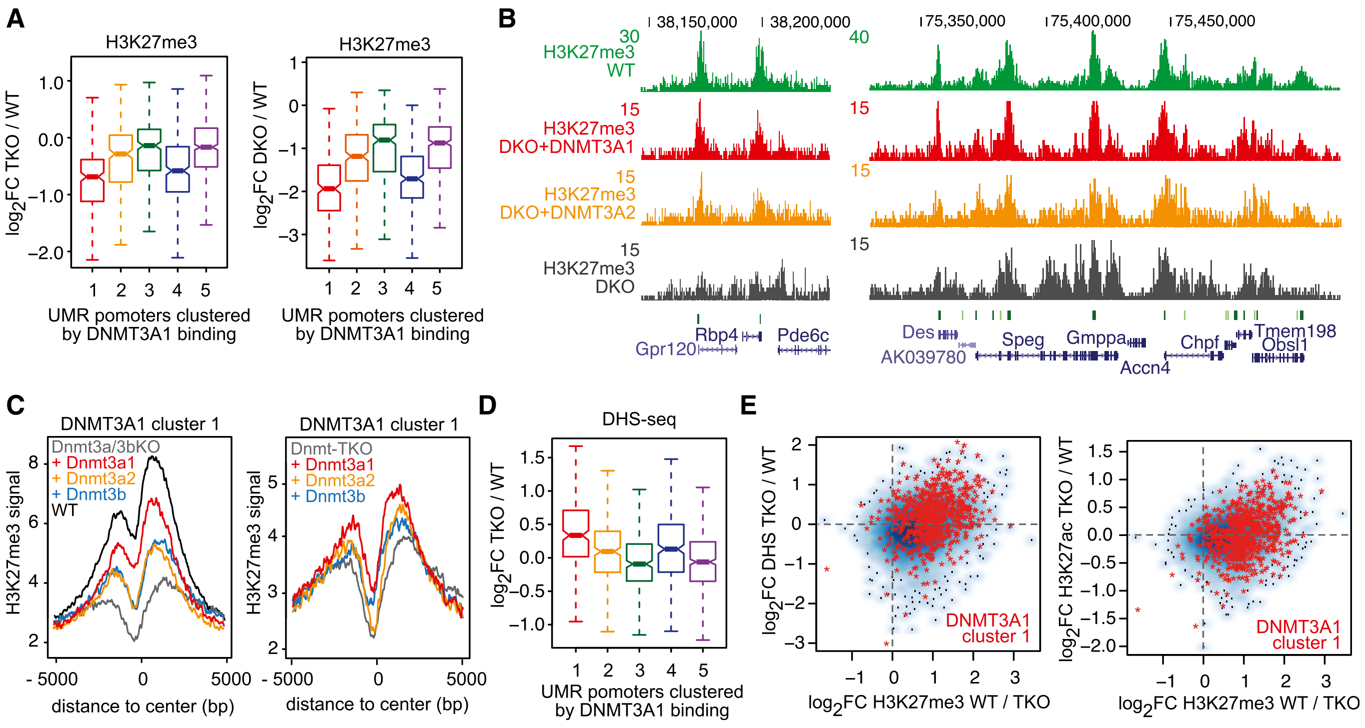

Figure EV3. Polycomb-regulated CpG island promoters bound by DNMT3A1 are frequently deregulated in the absence of DNA methylation.

A Box plots showing reduction in H3K27me3 in *Dnmt*-TKO and *Dnmt3a/Dnmt3b*-DKO cells at UMR promoters clustered based on DNMT3A1 enrichments (clustering based on Fig EV2A). Boxes denote the IQR and whiskers 1.5 × IQR. Notches display the confidence interval around the median.

B H3K27me3 reduction and spreading in *Dnmt3a/3b*-DKO cells is partially re-established upon re-expression of DNMT3A1. Genome browser examples for regions with dynamic H3K27me3 in *Dnmt*-TKO cells and *Dnmt*-DKO cells expressing DNMT3A1 or DNMT3A2. Shown are read counts per 100 bp.

C Average density plots showing library-normalized H3K27me3 ChIP-seq reads around UMR promoters bound by DNMT3A1 (cluster 1 from Fig EV2A) in *Dnmt*-TKO cells and *Dnmt*-TKO cells expressing individual *de novo* DNMTs.

D Box plot showing increased DNaseI hypersensitivity (DHS) signals in *Dnmt*-TKO cells at UMR promoters enriched by DNMT3A1 (same clusters as in Fig EV2A). Boxes denote the IQR and whiskers 1.5 × IQR. Notches display the confidence interval around the median.

E Scatter plots showing correlated increase in DHS or H3K27ac with loss of H3K27me3 at UMR promoters in *Dnmt*-TKO cells, suggesting that reduction in H3K27me3 leads to increased promoter accessibility and activity. Red dots denote the UMR promoters within cluster 1 bound by DNMT3A1.

Data information: Datasets used in (A–C) were obtained from King *et al* (2016). Datasets used in (D and E) were obtained from Domcke *et al* (2015).

© 2017 The Authors
